# Hydrogel Biocomposite of Alginate and Mucilage of *Opuntia ficus-indica* Cactus in the Adsorption of Methylene Blue in Aqueous Solution

**DOI:** 10.1021/acsomega.4c07325

**Published:** 2024-12-24

**Authors:** Estefane Caetano Nazzari, Gessica Wernke, Grace Anne Vieira Magalhães Ghiotto, Rosângela Bergamasco, Raquel Guttierres Gomes

**Affiliations:** † Department of Biotechnology, Genetics and Cell Biology, Biological Sciences Center, State University of Maringá, Maringá, Paraná 87020-900, Brazil; ‡ Department of Chemical Engineering, Technology Center, State University of Maringá, Maringá, Paraná 87020-900, Brazil; § Department of Food Engineering, Technology Center, State University of Maringá, Maringá, Paraná 87020-900, Brazil

## Abstract

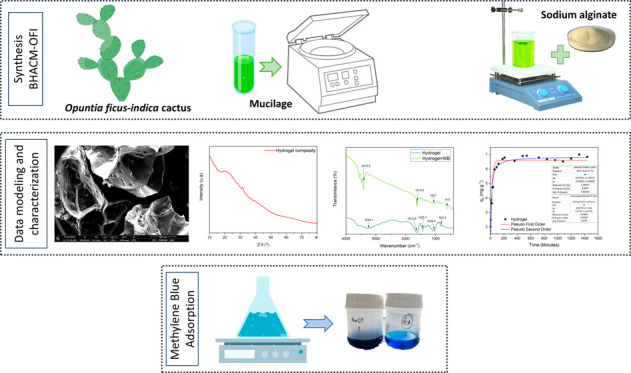

This work analyzes the production of a hydrogel composed of mucilage from the cactus *Opuntia ficus-indica* (OFI) and sodium alginate. In obtaining the new material, green synthesis was used, free of chemical compounds, and applied in the treatment of textile effluent for the adsorption of methylene blue (MB). The hydrogel was characterized by FT-IR, XRD, SEM, and zeta potential. The swelling study showed a maximum value of 262% at pH 6.0. Adsorption studies revealed a maximum adsorptive capacity of 7.21 mg g^–1^ in 400 min at 298 K. Furthermore, the experimental data showed better fit to the pseudo-second order and Langmuir models for kinetic and isothermal studies, respectively. The adsorptive process showed spontaneous and exothermic behavior as well as a chemisorptive nature. It is noteworthy that in the studies conducted at a higher concentration of the contaminant, the maximum adsorption was 760 mg g^–1^. The reuse of the hydrogel was effective for five cycles, maintaining the adsorption of approximately 50% MB removal. Therefore, the biodegradable hydrogel is a material that contributes to the environment, is low cost, with simple synthesis, and is a promising new material for large-scale applications, considering its sustainable character and high efficiency in the adsorption of MB in aqueous solution.

## Introduction

Correlated to the economic importance of the textile industry in Brazil is the treatment of effluents generated by this sector, which are difficult to treat and have low biodegradability, in addition to the solubility and residual color of the dyes, the effluent has recalcitrant characteristics, as well as the presence of bleaching agents, alkalis, acids, surfactants, and dispersants.^1^ In particular, dyes have a great polluting potential even at low concentrations, affecting the ability of photosynthetic organisms in the water bed, mainly related to light penetration, in addition to the impact of gas solubility, toxicity, and mortality of aquatic organisms, resulting in eutrophication processes.^2^

The main effluent treatment methods include the use of coagulation-flocculation, sedimentation, ion exchange, flotation, membrane separation, reverse osmosis, oxidation, and filtration; however, they are inefficient in removing contaminants within satisfactory disposal parameters or are onerous.^3^ In this way, seeking efficiency and low cost, new materials for application in the treatment of effluents are continuously developed,^4^ such as hydrogels. In addition to hydrogels, other low-cost materials have also stood out due to their efficiency in the adsorption process, such as activated carbon, zeolites, bentonite, chitosan, graphene, and agricultural residues, such as orange peel, sugar cane bagasse, and coconut. These materials, aside from being effective in the adsorption process, are highly available.^5,6^

Hydrogels are materials that have been successfully applied in the treatment of effluents due to their expansion properties, reuse capacity based on adsorption–desorption cycles, synchronous action in different functions, and easy removal of contaminants. In water treatment, hydrogels act simultaneously in the remediation of various contaminants, such as drugs, dyes, heavy metals, and inactivation of microorganisms.^7^ The synthesis of composite hydrogels, using more than one source of polymers, allows a varied application and stability of the material, also allowing the increase of adsorption efficiency when the addition of natural polymers incorporates functional groups, such as hydroxyls, carboxyls, and amino, which tend to increase the removal capacity of metal ions, dyes, and some organic contaminants, facilitating the interaction with these groups. In this sense, the use of nontoxic compounds, preventing secondary pollution from the treatment of effluents, is required.^8−11^

*Opuntia ficus-indica* (OFI), belonging to the Cactaceae family, is native to Latin America, South Africa, and the Mediterranean region.^12^ The Cactaceae have been widely researched in the cosmetics industry,^13,14^ medicine,^15^ pharmaceuticals,^16^ food,^17^ and water treatment,^18^ among others, due to the anti-inflammatory, antimicrobial properties,^19^ antioxidants, and the emulsifying, gelling, and thickening characteristics.^18^

Structurally, due to its elastic property and the ease of forming a continuous matrix, the mucilage present in OFI has been used in the production of resistant biosorbents for treating water and effluents,^20^ mainly related to the polymeric composition, known for its use in the treatment of effluents through adsorption and coagulation/flocculation processes, as for example in studies conducted by El Bouazzaoui et al.^21^ in the extraction of cellulose from the seed of *Opuntia ficus-indica* to remove the methylene blue dye.

Although some studies in the literature employ alginate^22,23^ or mucilage^24,25^ derived from various natural materials for the removal of contaminants in water, they are synthesized in isolation. However, this study stands out by incorporating both components into a single adsorbent material, forming a biodegradable hydrogel. The analysis of the adsorptive capacity of this composite material offers an innovative approach, with the potential to enhance the efficiency of contaminant removal, combining sustainability and effectiveness in water treatment.

Thus, considering the green and sustainable process of developing new materials, especially applied to water treatment, the objective of this study was to carry out the synthesis and characterization of a biodegradable hydrogel of alginate and OFI mucilage (BHACM-OFI), through green synthesis, aiming at the removal of methylene blue from aqueous media.

## Materials and Methods

### Mucilage Extraction

The mucilage was extracted from OFI cladodes using the aqueous part of the cactus, following the method proposed by Cruz-Rubio et al.,^26^ and Otálora et al.^27^ with some modifications, such as no use of organic solvents in the preparation of the gels, to guarantee a totally green synthesis. After cleaning the cladodes, to obtain the bioactives from the aqueous part, the pulp was cut and crushed in a blender, followed by centrifugation at 4000 rpm for 30 min and subsequent collection of the supernatant. The place where the cladodes were collected is located near the State University of Maringá (S 23° 24.532166’ e W 51° 56.231299’).

### Synthesis of Hydrogel Granules

To prepare the hydrogel granules, sodium alginate (90%, Dinâmica–BR) was diluted in deionized water and cactus mucilage, in a proportion of 1:40:40 (g: v: (v).

**Figure 1:**
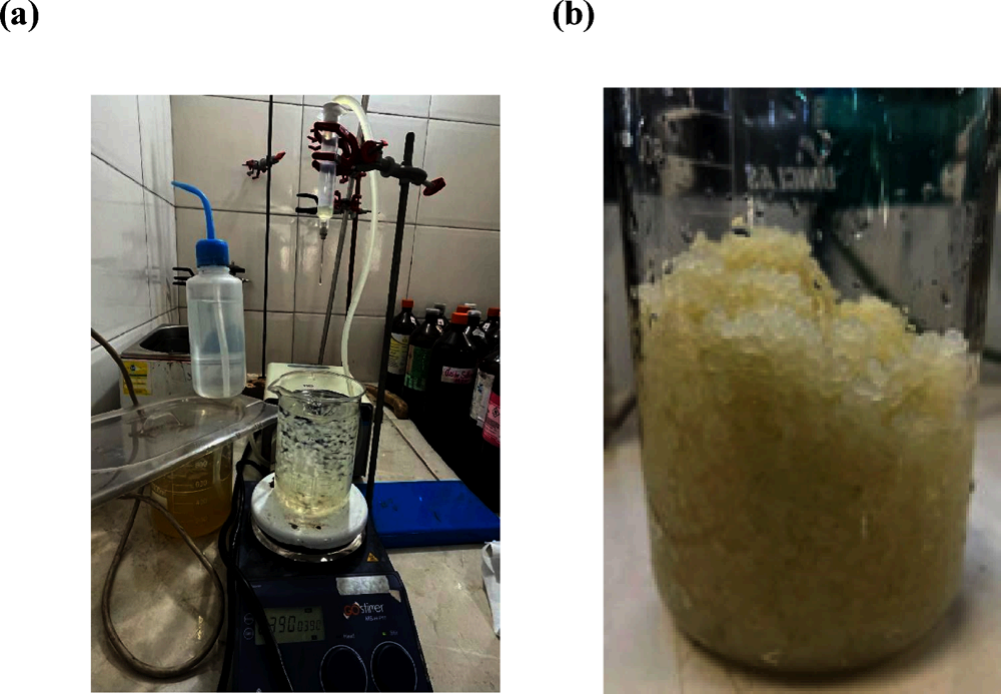
BHACM-OFI dripping in CaCl_2_ (a) and obtaining BHACM-OFI (b).

Then, the solution was subjected to a magnetic stirrer for incorporation and homogenization of the mixture, forming a gelatinous solution. For reticulation and formation of granules, a 10% concentration of calcium chloride solution was prepared, and the formulation was dripped on the solution (>99%, Anidrol - BR), which presents no toxicity and easy availability,^28^ as represented in Figure 1. After the formation of the granules, they were washed in deionized water to remove excess CaCl_2_ and lyophilized, to preserve the structure and guarantee mechanical resistance, in addition to increasing swelling rates.^29,30^

### Characterization of the Hydrogel

The hydrogel was characterized by using different techniques regarding its physical and chemical properties. The physical properties were investigated using scanning electron microscopy (SEM), X-ray diffraction (XRD), and zero charge point techniques. For the elucidation of the chemical structure, Fourier-transform infrared spectroscopy (FTIR) was used.

For such characterizations, the SEM analysis (SEM - Quanta FEI–250) was performed after metallization of the samples, with magnification varying between 150× and 10,000×. For the XRD analysis (DRX - ShimadzuLabX 6000 X-ray diffractometer), a voltage of 40 kV at 30 mA was used, with a rate of 2° θ/min^–1^ rate, an acquisition time of 1 s, using a time interval of 5° ≤ 2θ ≤ 60°. Analyses were performed using CuKα irradiation. The FTIR analyses were performed using an Avatar 360-FTIR device (Thermo Nicolet Instrument), and readings were taken between 400 and 4000 cm^–1^.

For the analysis of the surface charge (pH_pcz_) and the analysis of the swelling of the hydrogel, a sample with 0.05 g of hydrogel in 30 mL of deionized water was used and analyzed under different conditions of initial pH (2 a 11), according to the methodology described by Das et al.^31^ The pH was adjusted with the aid of a pH meter (Hanna) using 0.5 M NaOH and 1 M HCl solutions. Subsequently, the samples were left under orbital agitation (shaker) 150 rpm at a fixed temperature of 298 K for a period of 24 h. After this period, an aliquot of the supernatant was removed, and the pH of the solution was measured. A graph was created with the difference between the final and initial pH of the solution as a function of the initial pH, obtaining the pH_pcz_ value of the material corresponding to the point where the pH variation was equal to zero. The PCZ value was calculated from eq 1:
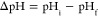
1where pH_i_ and pH_f_ represent the initial and final pH of the sample, respectively.

### Swelling Study

For the analysis of the swelling of the hydrogel after 24 h in an aqueous medium with 30 mL of deionized water, the excess surface water of the material was removed with a sieve and paper towel, and soon after, the adsorbent was weighed. Also, the analysis was carried out with pH variations between 2 and 11, with 0.5 M NaOH and 1 M HCl solutions. The swelling percentage was calculated using eq 2:
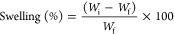
2where *W*_i_ is the initial dry weight and *W*_f_ is the final swollen weight of the adsorbent.

### Adsorption Study

The adsorption study was carried out in batches, first varying the mass (0.025 to 0.125 g), pH (4, 6, and 10), and ionic strength, adjusted with NaOH, HCl, CaCl_2_, MgCl_2_, NaCl, and KCl. To analyze the influence of pH on the removal capacity, the experimental results were evaluated using the analysis of variance (ANOVA) test. Kinetic and adsorption isotherm studies were also carried out. In these studies, dry hydrogel and flasks containing 30 mL of methylene blue solutions at a concentration of 20 mg L^–1^ were used.

The flasks were maintained under constant stirring at 150 rpm and a temperature of 298 K for 24 h. All analyses were performed in duplicate. The samples were separated using filter paper to separate the adsorbent and the solution; then, an aliquot of 5 mL was removed from each sample to carry out the readings of the initial and final concentrations of the contaminant, and the readings of the samples were established using a spectrophotometer UV–vis (HACH DR 500) at a wavelength of 662 nm. Removal efficiency (*R*%) and adsorption capacity (*q*_e_) were calculated using eqs 3 and 4, respectively.
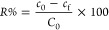
3
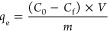
4where *C*_0_ is the initial concentration, *C*_f_ the final concentration, *m* is the mass of the adsorbent, and *V* is the volume of the solution.

The kinetic analysis of MB adsorption was carried out under the same conditions previously reported, at predetermined time intervals, and the pseudo-first-order (PFO) (eq 5) and pseudo-second-order (PSO) models were applied (eq 6).
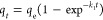
5
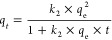
6where *q_t_* is the adsorption capacity (mg/g) at time *t*, *k*_1_ and *k*_2_ are the pseudo-first-order and pseudo-second-order adsorption constants, respectively, and *q*_e_ is the adsorption capacity at equilibrium. The analysis of the MB adsorption isotherm was performed by varying the concentrations from 50 to 65,000 mg L^–1^. To fit the experimental data, models were used with Langmuir (eq 7), Freundlich (eq 8), and Temkin (eq 9).
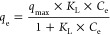
7
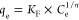
8
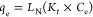
9where *q*_e_ is the adsorption capacity at equilibrium (μg/L); *C*_e_ is the equilibrium concentration of the residual substance in the solution; *K*_L_ and *K*_F_ are the Langmuir and Freundlich adsorption equilibrium constants, respectively; RL is the Langmuir separation factor; *n* is the Freundlich exponential factor; *C*_s_ is the monolayer formation concentration; *R* is the universal gas constant (8.314 J mol/K.); *T* is the temperature (K); AT is the Temkin binding constant (μg/L); and *b*_T_ is the Temkin isotherm constant (J/mol).

For a better understanding of the processes that govern adsorption, the thermodynamic parameters were calculated using Gibbs free energy, enthalpy, and entropy, according to the following eqs (eqs 10 and 11).
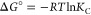
10where Δ*G*° is the Gibbs free energy (kJ mol^–1^), *R* is the ideal gas constant (8.314 J mol^–1^ K^–1^), *T* is the temperature (K), and *K*_C_ is the equilibrium constant.

The enthalpy and entropy are obtained from the parameters of the angular and linear coefficients of the graph ln *K*_C_ vs 1/*T*.
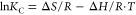
11where Δ*G*° corresponds to the Gibbs free energy, Δ*H*° corresponds to the enthalpy (kJ mol^–1^), Δ*S*° corresponds to the entropy (kJ mol^–1^ K^–1^), *T* is the temperature (K), and *K*_C_ is the equilibrium constant. Thus, *K*_C_ and *K*_L_ can be determined as described earlier.^32^

The adsorbent regeneration and reuse tests were carried out by successive cycles of adsorption–desorption for 24 h each, under the same conditions as before. The solutions used for the desorption study were 30 mL of water, HCl (0.1 M), NaOH (0.1 M), and ethyl alcohol.

## Results and Discussion

### Characterization of the Material

**Figure 2:**
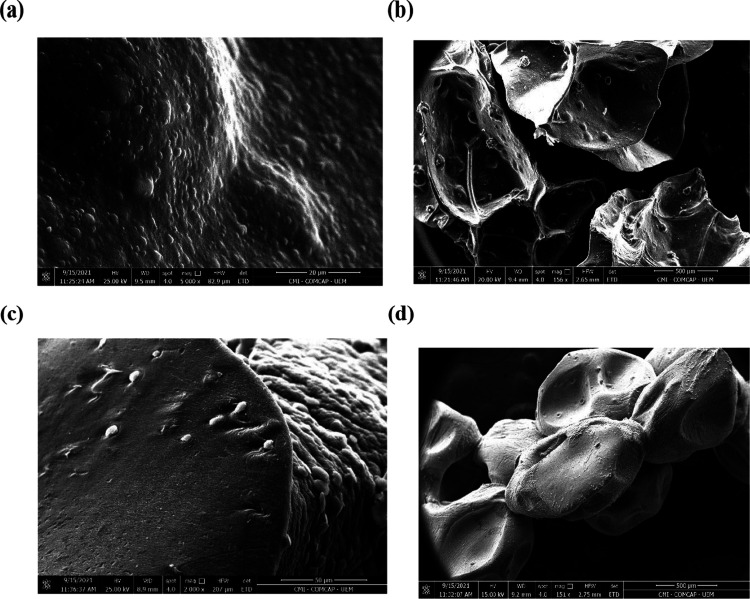
SEM analysis of the hydrogel synthesized from OFI cactus mucilage, using the freeze-drying method (a, b) and drying in an oven at 40 °C (c, d).

Figure 2 shows SEM images of the hydrogel formulated from OFI cactus mucilage. For the analysis of structural preservation, the hydrogel granules were lyophilized (Figure 2a,b) or dried in an oven at 40 °C (c and d).

Through the images presented in Figure 2, it can be observed that the samples that were lyophilized have fewer villi compared to those dried in an oven. Thus, Figure 2b,d shows the irregular surface of both samples as well as the presence of a greater number of cavities in the lyophilized sample. The porosity and cavity characteristics are desirable for adsorptive processes, since they increase the adsorption surface area available for adsorbate interaction.^18^ Furthermore, this drying method was standardized, taking into account the safety of storage as it deals with materials of organic origin.

**Figure 3:**
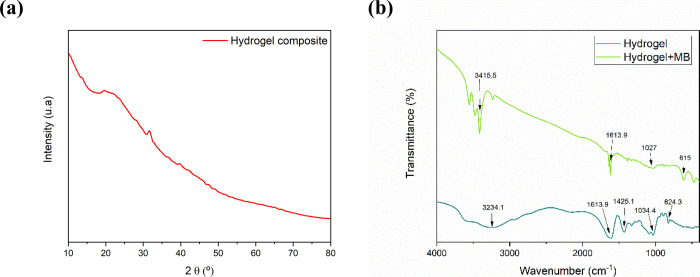
BHACM-OFI X-ray diffraction analysis (a) and BHACM-OFI FTIR analysis before and after MB absorption (b).

Based on the X-ray diffraction analysis (XRD), presented in Figure 3a, it can analyze the composition of the synthesized BHACM-OFI. The spectrum presents a material of low crystallinity and predominantly amorphous nature coming from the composition of alginate and OFI.^33^ According to Contreras-Padilha et al.,^34^ the presence of sodium oxalate associated with OFI maturation can be evidenced by the presence of the peak at 31°, as previously presented.^21,34,35^

The spectra obtained from the FTIR analysis demonstrate that the characteristics of the BHACM-OFI are evidenced by the presence of signals at 3234, 1613, 1425, 1034, and 824 cm^–1^. The broad band in the region of 3234 cm^–1^ corresponds to the OH group of alcohols and carboxylic acids present in both alginate and OFI.^18,34^ The presence of COO– bonds in ionized carboxylic acids, common in spectra from calcium oxalate, is commonly found in *Opuntia ficus-indica* cactus. In the same way, the signal could represent the deformation of primary amines NH_2_, which are identified by means of the 1613 signal cm^–1^ and are present in the cactus, and these may be the functional groups responsible for the interaction between alginate and OFI.^18,36^ The vibration present at 1425 cm^–1^ may be associated with vibration deformations of the C–H and OH groups of the phenolic groups.^12,37^ The peaks at 1034 and 824 cm^–1^ are associated, respectively, with the pyranose and CH_3_ ring vibration as well as the S=O stretching vibration.^38^

In the spectra of the adsorbent after the adsorption of MB, a slight deviation can be observed showing the crystallinity at the peak 3415 cm^–1^, which may correspond to the interaction of hydrogen bonds caused by the absorption of the contaminant. Likewise, the deviation from 1027 to 1034 cm^–1^ may still indicate the OFI pyranose ring and C–O–C or O–H vibrations,^34,39^ showing a possibility of π–π interactions acting on the contaminant removal.^40^ In the adsorbed contaminant spectrum, after adsorption, the signal disappears around 800 cm^–1^, and a new peak appears at 615 cm^–1^. According to ref (36), the absorption band at 800 cm^–1^ corresponds to C–H heteroaromatic and aromatic compounds in OFI compounds on the hydrogel. In the same way, Ovchinnikov et al.^41^ showed that signs on 615 cm^–1^ could be related to C–S–C functional groups, indicating monomers of ionic forms of MB^+^. Therefore, the changes in its signals in FTIR after adsorption are related to intermolecular interactions enrolled between methylene blue and the hydrogel in the adsorption process.

### PCZ and Swelling Study

The effect of swelling in gels is an advantageous feature and occurs due to the presence of ionic groups and the hydrophilic behavior, diffusing water into the gel network.^42^ The swelling of the polymer network increases the volume of the hydrogels, which facilitates both the exposure of adsorption sites and the diffusion of pollutants, making the adsorption process more efficient.^43^ In this sense, the interaction between the polymeric components of the hydrogel and the chemical species in the surrounding medium directly affects the swelling property, once the presence of ions in the solution can change the functional groups of the hydrogel, impacting the water absorption capacity.^44^

**Figure 4:**
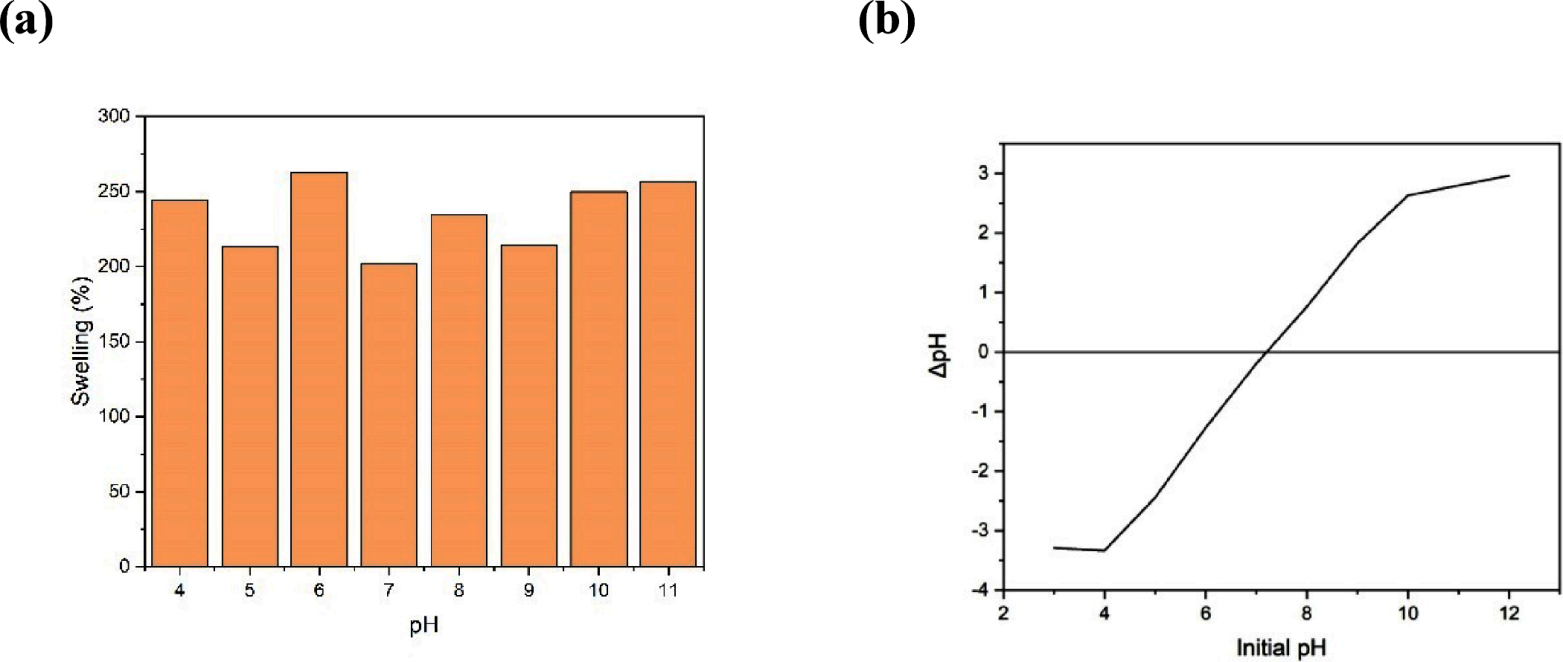
Swelling as a function of pH (a) and pH_pcz_ for BHACM-OFI (b).

The swelling study of the gels was carried out with a pH variation between 4 and 11, and it was observed that in all variations, there was swelling of the material. The maximum swelling was obtained at pH 6.0 with 262%, as shown in Figure 4.

In previous studies,^45−47^ it was observed that certain materials can be influenced by H^+^ ions at low pH, with increased interactions between the ions and the functional groups of the hydrogel, increasing cross-linking and decreasing swelling. The opposite was cited for basic pH. Such behavior was different from that found in the present material, which benefits the use of BHACM-OFI in neutral pH, facilitating the effluent treatment process, similar to studies conducted by Verma et al.^48^

The pH_pcz_ analysis can define adsorption capacity, ionization, pH dependency, and electrostatic interactions between the adsorbate and hydrogel granules.^49^ The adsorption process results in electrostatic interactions between the functional groups present in the MB and on the surface of the material.,^47^ such as the BHACM-OFI. For this purpose, zero charge point analysis (pH_pcz_) was performed. Thus, it is observed that when pH_i_ < pH_pcz_, the surface of the hydrogel becomes protonated, and when pH_i_ > pH_pcz_, the surface functional groups become negatively charged, resulting from deprotonation.^21,49^ In studies of adsorption by electrostatic interactions, it is expected that amine groups (−NH^2^) present on the surface of the material are protonated at acidic pH, causing repulsion of the contaminant.^31,39^

Figure 4b shows the zero charge point value of the adsorbent, around pH 7.2. Thus, for values below pH 7.2, the active sites of BHACM-OFI are positively charged, not favoring electrostatic interactions for the adsorption of the cationic MB dye. The same occurred in the studies of Verma et al.,^48^ using a hydrogel of sodium alginate modified with graphite, the surface of the hydrogel being positively charged did not help in the adsorption of the cationic dye used, which is malachite green.

In studies of the influence of pH variation on the adsorption process of methylene blue dye, the tests presented removal results of 6.46, 6.32, and 6.66 mg g^–1^ at pH of 4, 6, and 10, respectively. To determine the pH range with the highest adsorption capacity, analysis of variance (ANOVA) was used. The results show that the effect of pH on the dye adsorption process did not present a significant difference between the values, with a *p*-value of 1.0 and a standard deviation of 0.17, indicating low dispersion between the analyses.

Therefore, regarding the effects shown on swelling, the following tests were developed at pH 6, which was the natural scale of the dye in question. Thus, it is expected that electrostatic interaction, or physisorption, may not be the main mechanism responsible for the removal of cationic MB by the protonated hydrogel up to pH 7.2, as will be demonstrated subsequently, accompanied by kinetic and thermodynamic studies.

### Hydrogel Mass Effect

**Figure 5:**
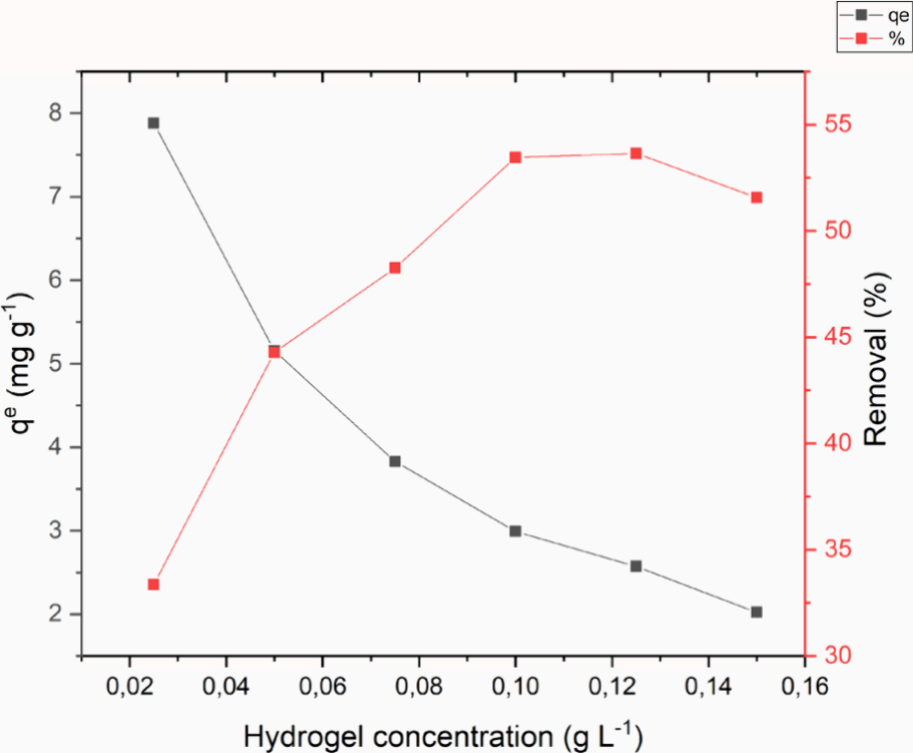
Study of the hydrogel mass effect on MB adsorption.

The effect of mass using BHACM-OFI as an adsorbent material is shown in Figure 5.

It is observed that the best MB removal efficiency occurred at the intersection between the best percentage of contaminant removal and the adsorptive capacity of the adsorbent (*q*_e_), represented by the hydrogel mass of 0.05 g, with an adsorption of 5.15 mg g^–1^.

This event can be explained due to the availability of free adsorptive sites for MB under osmotic pressure, and with the use of a lower dosage of adsorbent, the active sites are more used, increasing the adsorption capacity. On the contrary, with higher dosages of adsorbent, the adsorption reaches equilibrium faster due to osmotic pressure, even with more active sites available. Therefore, the ideal concentration selected for subsequent experiments was 0.05 g L^–1^.

This phenomenon can be attributed to the availability of free adsorptive sites for methylene blue dye (MB) under the influence of osmotic pressure. The use of smaller amounts of adsorbent results in more efficient utilization of active sites, which, in turn, increases the adsorption capacity. On the other hand, with higher dosages of adsorbent, the adsorption process reaches equilibrium more quickly due to osmotic pressure, even with the presence of a larger number of active sites.^21^ Based on these results, the optimal concentration of the adsorbent selected for subsequent experiments was 0.05 g L^–1^.

Thus, the choice of the optimal concentration of 0.05 g L^–1^, as presented, reflects a balance between the efficiency of active site utilization and the system’s saturation time. This approach optimizes the adsorption process by using a sufficient amount of adsorbent to ensure high adsorption capacity without wasting the material. Previous studies, such as those by Magalhães-Ghiotto et al.,^44^ demonstrated that optimizing the adsorbent dosage is essential to maximize the removal of contaminants from aqueous solutions in an economically viable and environmentally sustainable manner.

According to the presented results, reducing the amount of adsorbent led to more efficient use of active sites, which resulted in higher adsorption capacity. This can be explained by the saturation effect of available sites, where, with a smaller adsorbent mass, there is a higher rate of site occupation as osmotic pressure favors adsorption. This phenomenon is consistent with studies such as Husien et al.,^50^ which point out that in systems with low adsorbent dosages, efficiency per unit mass is maximized due to better utilization of available active sites, increasing the adsorption capacity per gram of the adsorbent material.

However, increasing the adsorbent mass reduces the concentration gradient between the adsorbent and the solution, which decreases the driving force for adsorbate molecule diffusion. This leads to a reduction in the adsorption rate and the rapid stabilization of the process.^51^

### Effect of Ionic Strength

Industrial effluents containing dyes contain several agents that interfere with the adsorption process. In order to analyze the ionic strength that can influence these systems, solutions of dissolved salts of CaCl_2_, MgCl_2_, NaCl, and KCl were used at concentrations of 0.1 and 0.3 mol L^–1^. As results obtained, it was evident that the adsorption capacity decreased for the various salts used as the concentrations increased from 0.1 to 0.3 mol L^–1^. Maximum *q*_e_ values of 1.5, 3.6, 2.3, and 1.7 mg g^–1^ for KCl, NaCl, MgCl_2_, and CaCl_2_ at 0.1 mol L^–1^, respectively, were observed. This occurs due to the competition between the active sites of the adsorbent, between the dye and the ions of the salts, both cationic, which shows the consequent electrostatic interaction in the removal of MB.^52^ Therefore, since the concentration of the dissolved salts is relatively low in real effluents, in industrial practice, the electrostatic interactions of the salts will have a low impact on the adsorptive process of this contaminant.^53^

The MB adsorption decreases as the salt concentration increases due to the increase in K^+^ and Na^+^ ions, showing that Na^+^ ions are more competitive with the active sites than K^+^. Such a behavior was observed in the study conducted by Rehman et al.,^42^ where electrostatic repulsion occurred between the adsorbent in the removal of crystal violet and Congo red dyes.

### Adsorption Kinetics

**Figure 6:**
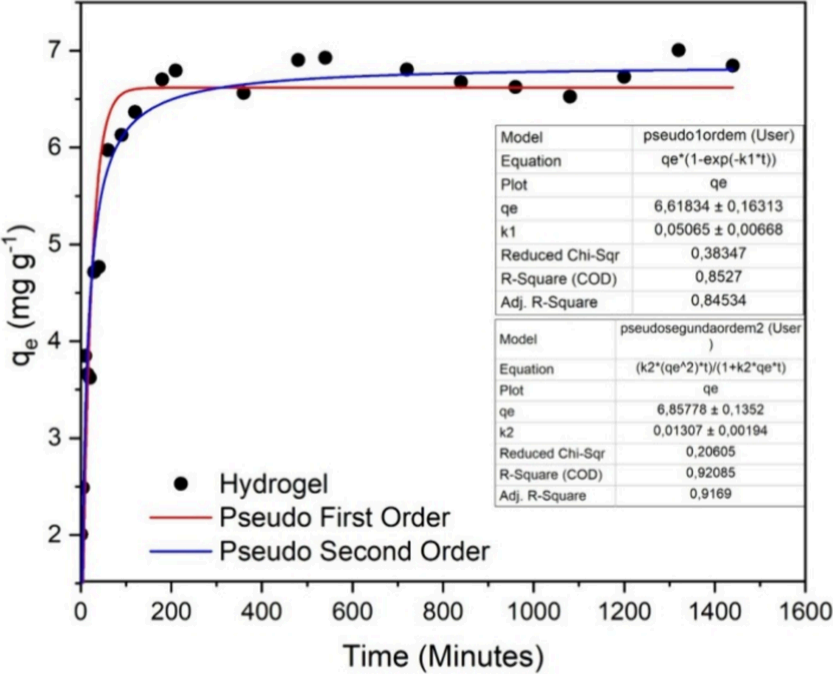
Adsorption kinetics using the BHACM-OFI.

In order to study the influence of contact time for dye removal by the synthesized hydrogel as well as the mechanisms involved in the adsorptive process, kinetic studies were performed. The adsorption kinetics experiments were investigated with time evaluations between 0 and 1440 min, using 0.05g of BHACM-OFI in 30 mL of MB at a concentration of 20 mg L^–1^ and at a temperature of 298 K. The adsorption data were analyzed by two kinetic models (pseudo-first and pseudo-second order) and are shown in Figure 6. Thus, the equilibrium time for the removal of MB and BHACM-OFI took approximately 400 min, with a maximum adsorption of 7.21 mg g^–1^.

The variation of the adsorption capacity in a kinetic graph reflects the dynamic process wherein rapid adsorption is initially due to the abundance of active sites in the adsorbent. As these sites fill up, the adsorption rate gradually decreases until reaching equilibrium, when the adsorption rate equals the desorption rate. As shown in Figure 6, between 800 and 1300 min of contact, the variation on qe behavior could be influenced by factors such as the external and internal diffusion of adsorbate molecules into the adsorbent, as well as by the progressive decrease in the concentration of the adsorbate in the solution, which reduces the efficiency of the adsorption process over time.^54^

According to the values shown for the correlation coefficient (*R*^2^ = 0.92), the model that best describes the kinetic behavior of adsorption is the pseudo-second-order model, assuming that adsorption is governed by chemisorption.^55^ The PSO model also fits in the study of Javanbakht and Shafiei^56^ with a hydrogel similar to that of the present study, however, using basil seed mucilage with sodium alginate, to remove Eriochrome black T dye.

### Equilibrium Isotherms

**Figure 7:**
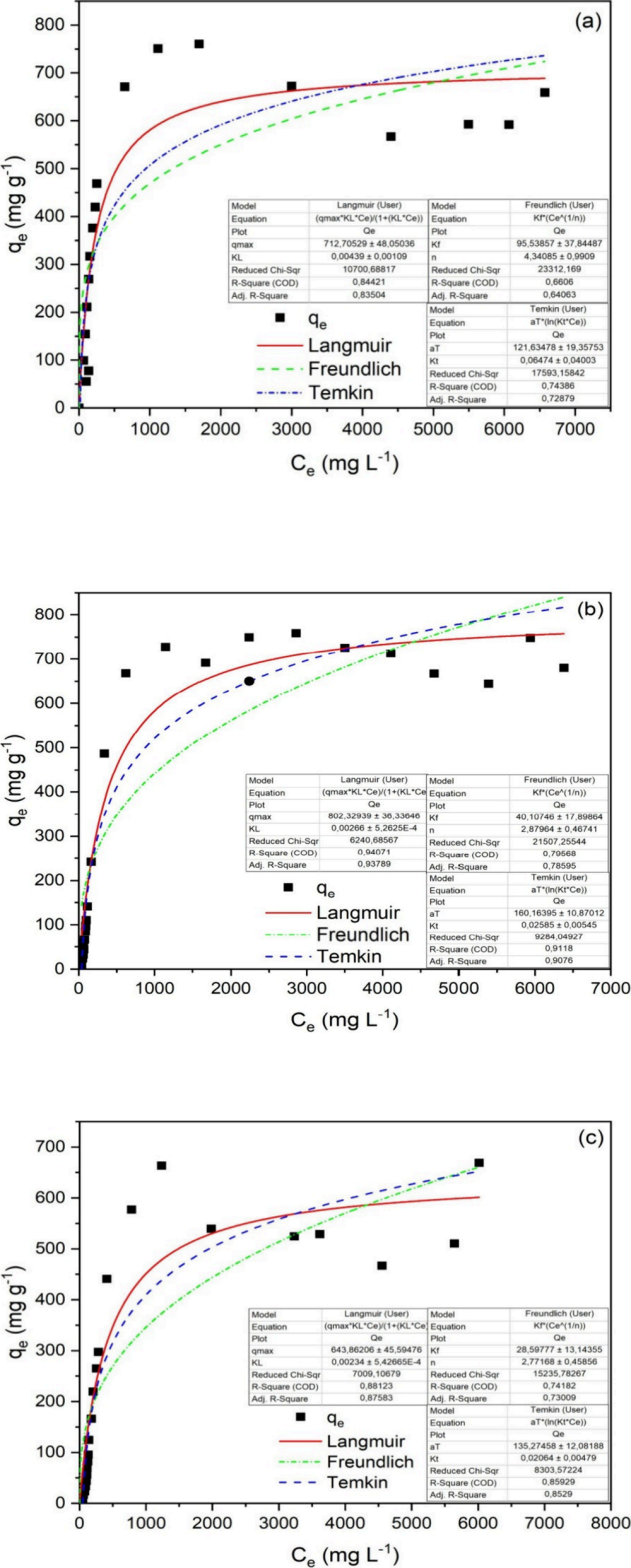
**MB** adsorption isotherm using BHACM-OFI at 298 (a), 308 (b), and 318 K (c).

The isotherm study allows evaluating the maximum adsorption capacity of a contaminant until material saturation, as well as the interaction between the adsorbent and the adsorbed species.^48^ To elucidate the distribution of MB molecules on the surface of BHACM-OFI, an equilibrium isotherm was used. Adopting the isothermal models of Langmuir, Freundlich, and Temkin, the equations, equilibrium data, and adjustment curves are shown in Figure 7.

As illustrated in Figure 7, the temperatures were evaluated at 298, 308, and 318 K, with the correlation coefficient values (*R*^2^ = 0.84, 0.94, and 0.88, respectively), where the best fit model occurred in the mathematical model of Langmuir, assuming that the removal of MB on the gel surface was performed in monolayers. In addition, the Langmuir isotherm model demonstrates that the material presented finite and identical sites in the removal process.^57^ In this way, the maximum adsorption capacity was obtained at 298 K with the removal of 760 mg g^–1^ of MB dye.

Such studies are similar to those found by El Bouazzaoui et al.,^21^ where an increase in temperature reduced the adsorption capacity. The optimal temperature for the adsorption of the MB dye with a similar hydrogel based on OFI was 298 K. Also, in a modified OFI hydrogel, Mahi et al.^39^ presented a Langmuir isotherm and removal of Orange-G dye by means of chemisorption.

Based on the temperature variation, in order to understand the energy stability, spontaneity, and nature of the sorption, thermodynamic studies were performed (Table 1).

**Table 1:** Thermodynamic Parameters for MB Removal Using BHACM-OFI

temperature (K)	Δ*G*° (kJ mol ^–1^)	Δ*H*° (kJ mol ^–1^)	Δ*S*° (kJ mol ^–1^ K ^–1^)
	
298	–2.5825	–76.6683	0.2501
308	1.3385		
318	2.35725		

According to the data presented in Table 1, through the studies of temperature variation and the concentration of the contaminant, the removal of MB through the BHACM-OFI showed an exothermic process with a chemisorptive nature (Δ*H*° = −76.67) and with reversibility propensity entropy and random adsorption (Δ*S*° = 0.25). The negative value for Δ*G*° at 298 K predicts a spontaneous and feasible interaction, unlike the values found at 308 and 318 K, demonstrated by the highest adsorption capacity at 298 K. The findings are similar to those found in the literature, with a viable, spontaneous, and reversible adsorption nature for the application of malachite green and OFI adsorbent,^58^ as well as adsorption process of exothermic nature, showing better removal at 298 K.^21^

### Regeneration Studies

The reuse of adsorbents needs to be economically and environmentally viable, since for industries, the effluent treatment process must have potential for large-scale applicability.^59^ Thus, desorption studies were carried out with the solvents HCl 0.1 M, NaOH 0.1 M, and ethyl alcohol P.A. The efficiencies of the desorption were 47.78, 46.74, and 47.07% for water, HCl, NaOH, and alcohol, respectively. This result can be explained by the interaction between the desorbent solution and the material’s adsorption sites, rich in NH2, which, due to protonation from exposure to H^+^ ions, may have generated electrostatic repulsion of the contaminant, thus facilitating the regeneration of the hydrogel.^60^ Since HCl was the best solvent observed in MB desorption, it was used in consecutive tests, in agitation cycles of 1440 min each over five cycles of adsorption–desorption. After the reported cycles, the hydrogel maintained the same initial adsorption capacity, with an approximate *q*_e_ of 7 mg g^–1^, proving to be suitable for reuse.

Similar studies also show that acidic solutions have been shown to be adequate in mechanisms for the reuse of hydrogels, as in the case of BHACM-OFI, making it a gel capable of being reused and recycled, becoming an advantageous option from both an economic and an economic point of view.^61^

Additionally, the HCl regeneration process, utilizing a relatively small 30 mL solution volume, offers a distinct advantage in terms of efficiency. It requires significantly less solution to regenerate the material compared to the initial volume of methylene blue-containing solution treated, considering the maximal adsorption capacity, as shown by isotherm studies. This approach enables the recovery and reuse of the contaminant and showcases the desorption process’s cost-effectiveness, speed, high efficacy, and environmental sustainability.^62^

## Conclusions

Hydrogels produced by green synthesis with OFI mucilage and alginate were used to remove methylene blue dye from aqueous solutions. They presented a rough surface, with predominantly amorphous material, with evidence of the presence of sodium oxalate in the composition, as well as the presence of functional groups such as ionized carboxylic acids, hydroxyls, phenolic, and nitrogen groups on the surface, which are responsible for the adsorption interactions with the contaminants. The material showed a neutral charge at pH 7.2, with a predominantly positive charge at acidic pH, as used in the study, approximately at pH 6.0. The study of the degree of swelling showed a maximum swelling of 262% at pH 6.0.

In the batch adsorption tests, the adsorption capacity was 7.21 mg g^–1^ in 400 min of testing, with the use of 0.05 g of adsorbent, corresponding to the intermediate mass tested. In the study of the influence of ions on the adsorption process, there was a decrease in adsorption, inversely proportional to the addition of salts. The kinetic data were better adjusted to the pseudo-second-order equation, as well as the Langmuir model for the isothermal studies, with better performance at 298 K, with a maximum adsorption of 760 mg g^–1^ of MB dye in monolayers. The thermodynamic study showed a spontaneous process (negative Δ*G*° at 298 K), with a chemisorptive and exothermic nature (Δ*H*° = −76.67), which is random and reversible. In the desorption study, the best performance was found in the 0.1 M HCl solution, with an approximate adsorption capacity of 7 mg g^–1^ in reuse for five cycles.
